# Exploring the *Cunninghamia lanceolata*(Lamb.) Hook Genome by BAC Sequencing

**DOI:** 10.3389/fbioe.2022.854130

**Published:** 2022-03-07

**Authors:** Yuan Ji, Liming Zhu, Zhaodong Hao, Shunde Su, Xueyan Zheng, Jisen Shi, Renhua Zheng, Jinhui Chen

**Affiliations:** ^1^ Key Laboratory of Forest Genetics & Biotechnology of Ministry of Education, Co-Innovation Center for Sustainable Forestry in Southern China, Nanjing Forestry University, Nanjing, China; ^2^ School of Life Science, Huaiyin Normal University, Jiangsu, China; ^3^ The Key Laboratory of Timber Forest Breeding and Cultivation for Mountainous Areas in Southern China, Fujian Academy of Forestry, Fuzhou, China; ^4^ National Germplasm Bank of Chinese Fir at Fujian Yangkou Forest Farm, Shunchang, China

**Keywords:** BAC, *Cunninghamia lanceolata* (lamb.), genome, Chinese fir, China

## Abstract

*Cunninghamia lanceolata* (Lamb.)Hook is an important economic timber tree in China. However, its genome characteristics have not been extensively assessed. To better understand its genome information, the bacterial artificial chromosome (BAC) library of chinese fir was constructed. A total of 422 BAC clones were selected and divided into 10 pools and sequenced, and with an average insert size of 121 kb, ranging from 97 to 145 kb. A total of 61,902,523 bp of reference sequences were sequenced and assembled, and based on an estimated genome size of 11.6 Gb for Chinese fir, the BAC library was estimated to have a total coverage of 0.53% genome equivalents. Bioinformatics analyses were also performed for repeated sequences, tRNAs, coding gene prediction, and functional annotation. The results of this study provide insights into the brief structure of the Chinese fir genome and has generated gene data that will facilitate molecular investigations on the mechanisms underlying tree growth.

## 1 Introduction

*Cunninghamia lanceolata* (Lamb.) Hook, also known as Chinese fir, is one of the most economically important indigenous coniferous species in south China (Li et al., 2017). It is considered an economic timber species because of its fast growth, good material, strong wood, hardiness, versatile use and high timber yield per unit, which is used extensively for pulp, paper, and lumber (Hu et al., 2017; Zhu et al., 2017), and thus has gained the interest of researchers. Since the 1960s, systematic genetic improvements have been carried out in Chinese fir. Improvement of Chinese fir through conventional breeding approaches is difficult and time-consuming due to its long generation time, large genome, and highly heterozygous genetic background (Hu et al., 2017). However, improved understanding of the Chinese fir genome may facilitate tree improvement by means of molecular marker-assisted breeding and genetic engineering. The extremely large size and complexity of the Chinese fir genome has presented challenges for its characterization. Despite these challenges, Chinese fir genomic resources such as sequence-related amplified polymorphism (SRAP) (Wang et al., 2014), single nucleotide polymorphisms (SNPs) (Zheng et al., 2019), amplified fragment length polymorphism (AFLP) (Tang et al., 2008), expressed sequence tags (ESTs) (Wang et al., 2007a), simple sequence repeat (SSR) (Lin et al., 2020), cp genome sequence (Zheng et al., 2016), and transcriptome data are available (Zhang et al., 2016). For example, SRAP markers were used to assess genomic variability among 327 Chinese fir genotypes and preliminarily identified 163 significant marker-trait associations for mature growth and wood property traits (Zheng et al., 2015; Dharanishanthi and Ghosh Dasgupta, 2018). The genetic diversity and relationships of 150 Chinese fir genotypes were investigated using morphological analysis and sequence-related amplified polymorphism markers. Some researchers used SLAF-seq for conifer genomic analysis, and at least of 147,376 SNPs were detected using EcoRV-based DNA SLAF-seq (Duan et al., 2016; Su et al., 2016). Approximately 195 AFLP markers were used to construct genetic linkage maps in Chinese fir (Tong and Si, 2004). Transcriptome analysis was employed to study wood formation in Chinese fir, and 405 unique ESTs were obtained by combining the construction of forward and reverse subtracted libraries using SSH with transcript profiling of clones using macroarray and real-time PCR (Wang et al., 2007b), and 30 SSR markers were developed to evaluate the genetic diversity of 94 samples of three Cunninghamia lanceolata populations.

Bacterial artificial chromosome (BAC) libraries carrying large insert genomic DNA are essential genomic resources for map-based cloning, physical mapping, genome sequencing, comparative genome research, chromosome location, and molecular marker development. Several BAC libraries have been reported for different conifers, which include those from Pinus taeda L (Magbanua et al., 2011; Magwanga et al., 2019), Picea glauca (Hamberger et al., 2009), and bald cypress (Liu et al., 2011). Based on these resources, researches on genome characterization, gene isolation, and targeted cloning have been conducted.

In this study, the BAC library of Chinese fir was established to identify genes and assess their distribution. The objectives of the present study were to sequence, assemble, and annotate 422 BAC clones randomly to explore Chinese fir gene distribution and identify genes responsible for Chinese fir adaptive and economic traits. By repeated sequence identification, 40,669,585 bp of repeats were identified, accounting for 65.7% of the sequence coverage. According to the KEGG, KOG, and GO database annotation comparisons, a total of 637 protein-coding genes were annotated and a total of 34,519 SSR loci were searched. At the same time, we designed random primers to verify that the average accuracy of the BAC sequence is high, which proves the credibility of the BAC sequence in this study.

The results of this study provide insights into the overall structure of the Chinese fir genome while generating gene data that may facilitate molecular investigations on mechanisms underlying tree growth.

## 2 Materials and Methods

### 2.1 BAC Library Construction

#### 2.1.1 Preparation of High-Molecular Weight Chinese Fir DNA

The leaves of a Chinese fir named 6,421 planted in Yangkou forest farm, Fujian, China were collected for DNA extraction. The isolation of Chinese fir nuclei was performed as described elsewhere (Zhang et al., 1995). The main experimental steps were obtained by sucrose density-gradient centrifugation, encapsulated into low-melting point agarose, and obtained its DNA by digestion with protease K. Approximately 20 g of etiolated leaves were ground in liquid nitrogen until fine powder was obtained, then transferred to a flask containing nuclear extraction bufferII (10 mM Tris, 100mM KCl, 10 mM EDTA·2 Na, 500 mM sucrose, 4 mM spermidine, 1 mM spermine, 0.10% (w/v) ascorbic acid, 0.13% (w/v) sodium citrate, 0.15% (v/v) β-mercaptoethanol, pH 9.45), and stirred on ice for about 20 min. The samples were filtered through four layers of gauze and one layer of cotton cloth. The filtrates was mixed with nuclear extract II (1/10 volume, 20.00% v/vTritonX-100) for 10 min, centrifuged at 4°C for 4,500 rpm for 20 min, then the supernatant was discarded and the pellet was isolated. Soft suspension sedimentation of brush, addition of nuclear extraction buffer II and centrifugation under the same conditions to collect the precipitate. The resulting pellet was washed with two additional solutions. First with nuclear extraction bufferII thrice, followed by another three washes using nuclear extraction buffer I (10 mM Tris, 100 mM KCl, 10 mM EDTA·2 Na, 500 mM sucrose, 4 mM spermidine, 1 mM spermine, 0.10% (w/v) ascorbic acid, 0.13% (w/v) sodium citrate, and pH = 9.45). The suspension nuclei were stored in 2 ml nuclear extract I at 45°C. The resulting pellet was resuspended in 2 ml of nuclear extraction buffer I at 45°C and gently mixed with an equal volume of 1.50% low-melting temperature agarose (nuclear extraction buffer I). The mixture was allowed to solidify after transferring to plug molds. The plugs were transferred into a proteinaseK solution (1 mg/ml proteinaseK, 1% (w/v) dodecyl sarcosine sodium, 0.1% ascorbic acid, 6 mM EGTA, 200 mM L-lysine, 0.13% (w/v) sodium citrate, and pH = 9.1) and incubated in a hybridization oven at 50°C with a gentle rotation for 48 h, then rewashed in four additional solutions. First, one time with 500 mM EDTA·2Na (pH9.1) and 50 mM EDTA·2Na (pH = 8.0). This was followed by three washeswith1 mg/mL PMSF (dissolved in anhydrous ethanol, diluted with TE, and pH8.0) and storage solution (20 mM Tris, 50 mM EDTA·2Na, and pH = 8.0), each timefor about 1 h at room temperature with gentle shaking. Finally, the plugswere stored in a storage solution at 4°C or immediately use dinenzyme digestion.

#### 2.1.2 Restriction Digestion of HMW DNA and Isolation of Size-Selected Fragments

Three DNA plugs were used to establish optimal HindIII partial digestion conditions. For partial digestion, 10 DNA plugs were divided into 40 pieces using a glass cover slip. The 38 pieces were digested in optimized conditions, and the remaining two pieces were used as negative and positive controls, i.e., without enzyme addition and excessive enzyme addition, respectively. Digested samples wereloaded into 1% agarose gel and subjected to pulsed-field gel electrophoresis (PFGE). DNA was visualized, and agarose fragments containing specific DNA sizes were cut from the gel slabs. A second and third PFGE run of the fragments was performed to further purify the DNA and remove small DNA fragments. After the third size selection, the recovered large DNAfragments were connected at 16°C for 24 h, then at 4°C for 48 h and converted. The clones were randomly selected and cultured in 3 ml of LB liquid medium containing 12.5 μg/ml chloramphenicol for 16 h. The plasmids were extracted by alkaline lysis (Bimboim and Doly, 1979). The plasmid insert fragment was released by NotI digestion and separated by pulsed field electrophoresis. The size of the inserted fragment was predicted using a low-range PFG marker. The sum of all the bands in a swim lane except the vector was the size of the cloned insert fragment, and no other band except the vector was regarded as an empty clone.

### 2.2 BAC Sequencing and Assembly

The original sequences of the BAC clones were determined by Illumina-X Ten and a PacBio high-throughput platform. For Illumina sequencing, plasmid DNA was extracted from10 BAC pools after quantitative culture of every 50 BAC clones. For each pool, the Illumina paired-end sequencing library with an insert size of 350 bp was constructed, and two-terminal sequence reading of 150 bp fragments was conducted to obtain the original sequence data of 150 Gb, with an average of about 15 Gb per pool.

For PacBio sequencing, the 10 BAC pools were further equally mixed into super pools to construct a PacBio sequencing library with insert size of 8–10 kb. The original sequence data of Illumina and PacBio were mixed and assembled to obtain the complete genome map of each BAC. First, Illumina raw sequence data were used to evaluate the complexity of sequences in each BAC pool. Velvet (v1.2.09) was used to assemble each BAC pool sequence and to preserve overlapping group sequences whose length is greater than or equal to 200 bp. Then, All Paths-LG and other de-Bruijn-based sequence splicing methods and PacBioToCA and other base correction methods in Celera software were used to complete the assembly of each BAC genome by mixed splicing. Finally, the constructed library was manually checked to ensure the correctness of each cyclic BAC sequence.

### 2.3 Bioinformatics Analysis

Snapgene software was used to convert nucl format to fasta format. VecScreen was employed to remove nucleotide sequences that may be of vector origin (Tang et al., 2015). Marker software was used for integrated analysis of protein-coding and non-coding genes (Cantarel et al., 2008). MITE-Hunter was employed *de novo* identification of miniature inverted-repeat transposable elements (MITEs) (Han and Wessler, 2010), Repeat Modeler was used to integrate LTR annotations (Flynn et al., 2020), and the homology-based repeat identification tool Repeat Masker was applied to identify repeats in Chinese fir BACs by comparing the viridiplantae section of the Repbase repeat database.

The GeneMark-ES (Lomsadze et al., 2005), Augustus (Hoff and Stanke, 2019), and SNAP software were used to predict protein-coding genes, and gene homology annotation was performed using the protein sequences of Universal Protein Resource (UniProt) database (http://www.uniprot.org). Protein-coding genes and noncoding genes were subjected to integrated analysis by Marker software. The Sprot and Phytozome databases were used for protein functional annotation. Gene sequences were aligned and classified by KOG database (Clusters of Orthologous Groups of proteins http://www.ncbi.nlm.nih.gov/COG/KOG). Pathway assignments were conducted according to the Kyoto Encyclopediaof Genes and Genomes pathway database (KEGG, http://www.genome.jp/kegg). For SSR locus statistical analysis, MISA script (https://webblast.ipk-gatersleben.de/misa/) was used to identify, define, and count SSR loci in the filtered data.

### 2.4 BAC Library Validation

Sequences spliced from pools 1 to 6 were selected as target sequences. Oligo7 software (v7.60) was used to design random primers with amplification lengths ranging from 500 to 800 bp, and 10 pairs of primers were designed for each pool. The amplified PCR products were Sanger sequenced and verified by BLAST (https://blast.ncbi.nlm.nih.gov/Blast.cgi).

## 3 Results

### 3.1 BAC Library Construction and Characterization

**FIGURE 1:**
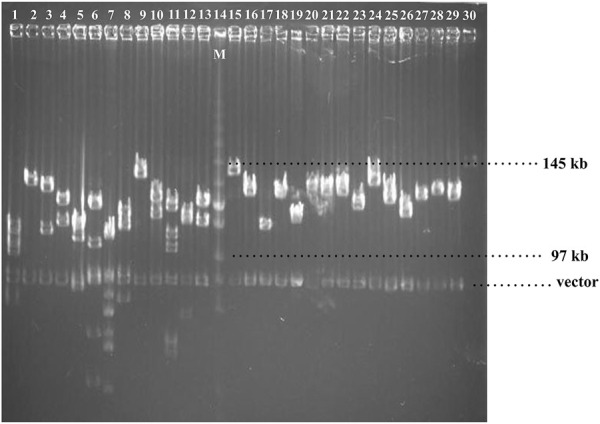
Pulsed-field gel electrophoresis of randomly selected. clones from Chinese fir BAC library.

**FIGURE 2:**
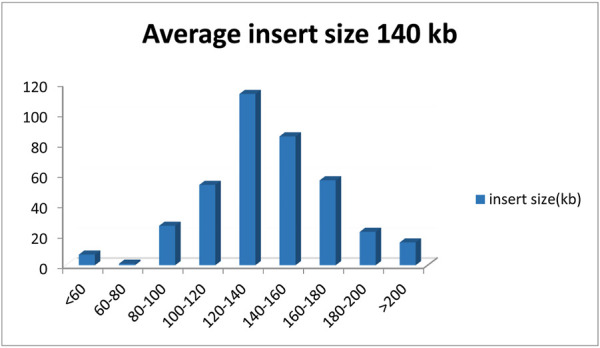
Distribution of the insert fragments from the Chinese fir BAC library.

A BAC library of Chinese fir 6,421 was constructed by partial digestion of total genomic DNA with HindIII and linked in a plndigo BAC-5 vector. The library contains 422 clones in 384-well microtiter plates. To examine the quality of the BAC library, 28 BAC clones were randomly selected, digested with NotI torelease the inserts from the plndigoBAC-5 vector, and analyzed by PFGE. The estimated average insert size was 121 kb, ranging from 97 to 145 kb, and no empty carrier were found (Figures 1, 2).

A total of 422 BAC clones were sequenced, and the average size was 1,46,688 bp, the total size was 61,902,523 bp, the minimal size was 21,158 bp, and the maximal size was 2,87,835 bp. GC content was 37.27% (Table 1). Considering the Chinese fir genome size (11.6 Gb) (Lin et al., 2020), the number of clones, and the average insert size, we estimate that the genome coverage of the library was about 0.53% genome equivalents.

**TABLE 1:** Summary of sequenced Chinese fir BAC clones.

BAC	Number
Number of BAC sequences	422
Mean base count (bp)	146,688
Total base count (bp)	61,902,523
Maximum BAC length (bp)	287,835
Minimum BAC length (bp)	21,158

### 3.2 Repetitive sequence Identification

The Chinese fir genome is rich in repeats. The masking results suggest that 21.32% of the nucleotides in the BACs. This low percentage of repeats is not particularly surprising as this has also been reported during repeat characterization in pine DNA (Wang et al., 2013). Homology-based methods yielded an estimated repeat content of only 27% for LP; it was not until initio repeat identification approaches were utilized that the repeat percentage increased to above 80%. Analysis using RepeatMasker identified 27,270 repeats in the Chinese fir BAC sequences, and the total interspersed repeats length was 40,669,585 bp, with 65.7% sequence coverage (Table 2). We found that long terminal repeats (LTRs) are the predominant repeat type in Chinese fir (47.67%) with members of the Gypsy LTR superfamily being the most abundant.

**TABLE 2:** Number and length of repeats in Chinese fir BAC.

Class	Superfamily	No	Length	Percentage
SINEs		2	74 bp	0%
	ALUs	0	0 bp	0%
	MIRs	1	12 bp	0%
LINEs		572	510,911 bp	0.83%
	LINE1	358	332,767 bp	0.54%
	LINE2	14	1,612 bp	0%
	L3/CR1	2	120 bp	0%
LTR elements		18,496	29,511,274 bp	47.67%
	ERVL	0	0 bp	0%
	ERVL-MaLRs	0	0 bp	0%
	ERV_classI	0	0 bp	0%
	ERV_classII	241	331,347 bp	0.54%
DNA elements		693	716,829 bp	1.16%
	hAT-Charlie	2	13 bp	0%
	TcMar-Tigger	0	0 bp	0%
Unclassified		14,951	9,930,497 bp	16.04%
Totalinterspersed repeats			4,0,669,585 bp	65.7%
Small RNAs		7	4,443 bp	0.01%
Satellites		15	3,028 bp	0%
Simplerepeats		7,110	6,52,511 bp	1.05%
Lowcomplexity		1,450	1,01,852 bp	0.16%

Sequences based on RepeatMasker analysis.

### 3.3 Non-coding RNA Prediction

A total of 55 transfer RNAs were predicted by tRNAscan-SE. Further analysis showed that eight of these were undetermined or unknown isotypes. Thirty tRNAs were located in the sense strand and 25 tRNAs were localized to the antisense strand of the genome sequence. Among these, tRNA gene encoding Ser (25.45%) predominated, followed by tRNA gene encoding Val (9.09%), and Gly (9.09%). Another 78 non-coding RNAs were predicted by Erpin, of which 57 were small nucleolar RNAs (snoRNAs).

### 3.4 Functional annotation and Classification

**FIGURE 3:**
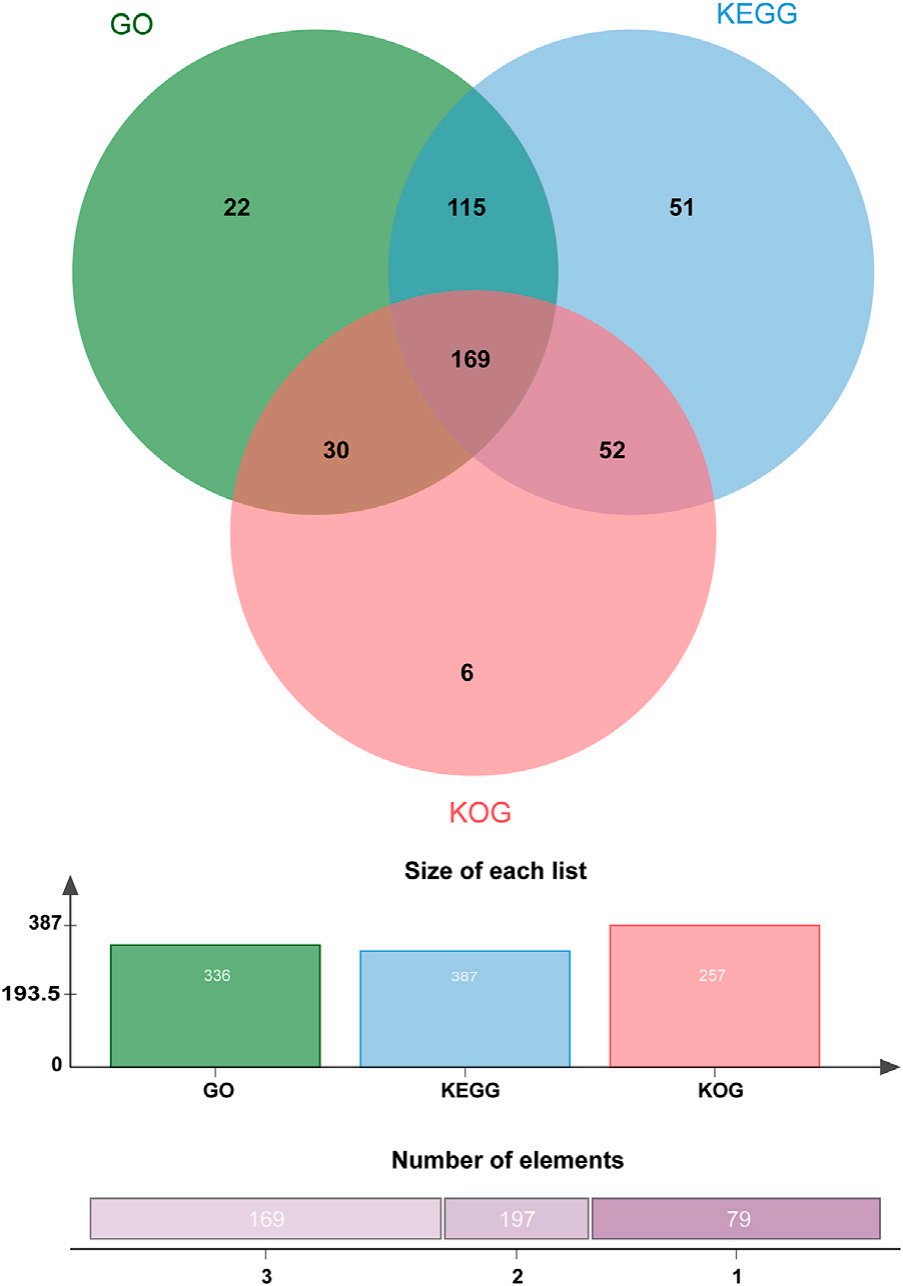
Venn diagram of the number of proteins annotated against public protein databases. A total of 445 proteins were differentially expressed, of which 336, 257, and 387 genes were functionally annotated using GO, KOG, and KEGG, respectively. Around 169 tags were shared by the three groups.

Approximately 445 of the 637 protein-encoding genes were annotated by Blastp against three public databases (KEGG, KOG, and GO). According to the number and proportion of genes in the different databases, there were differences in the number of genes annotated successfully because these databases use different filter conditions such as protein function and metabolic pathways involved (Figure 3).

**FIGURE 4:**
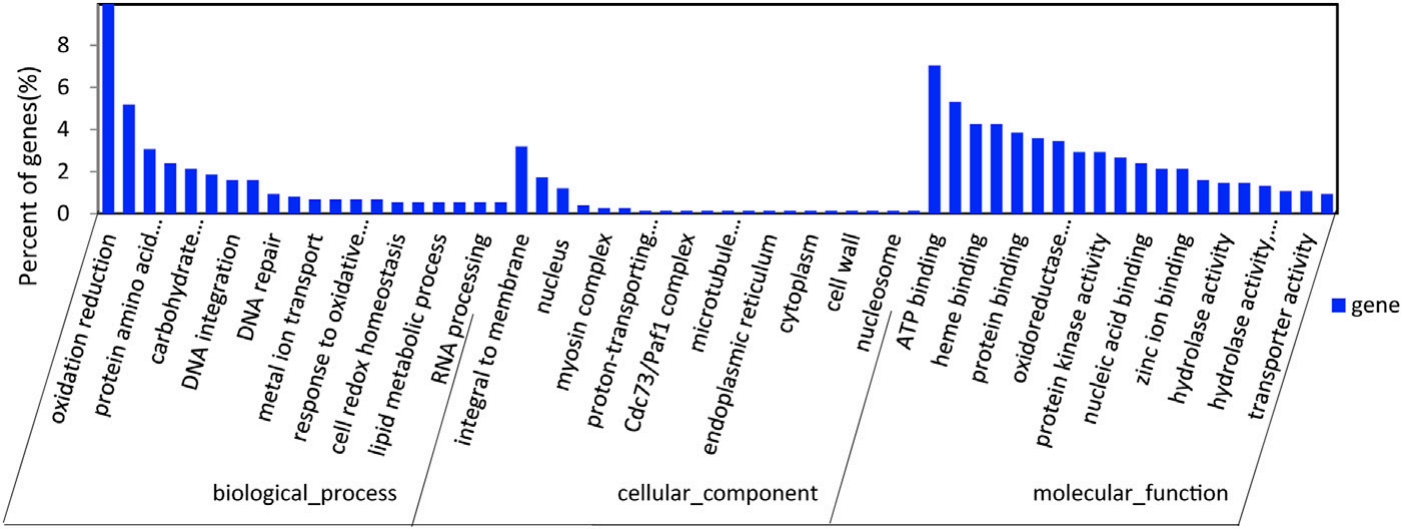
GO classification.

**FIGURE 5:**
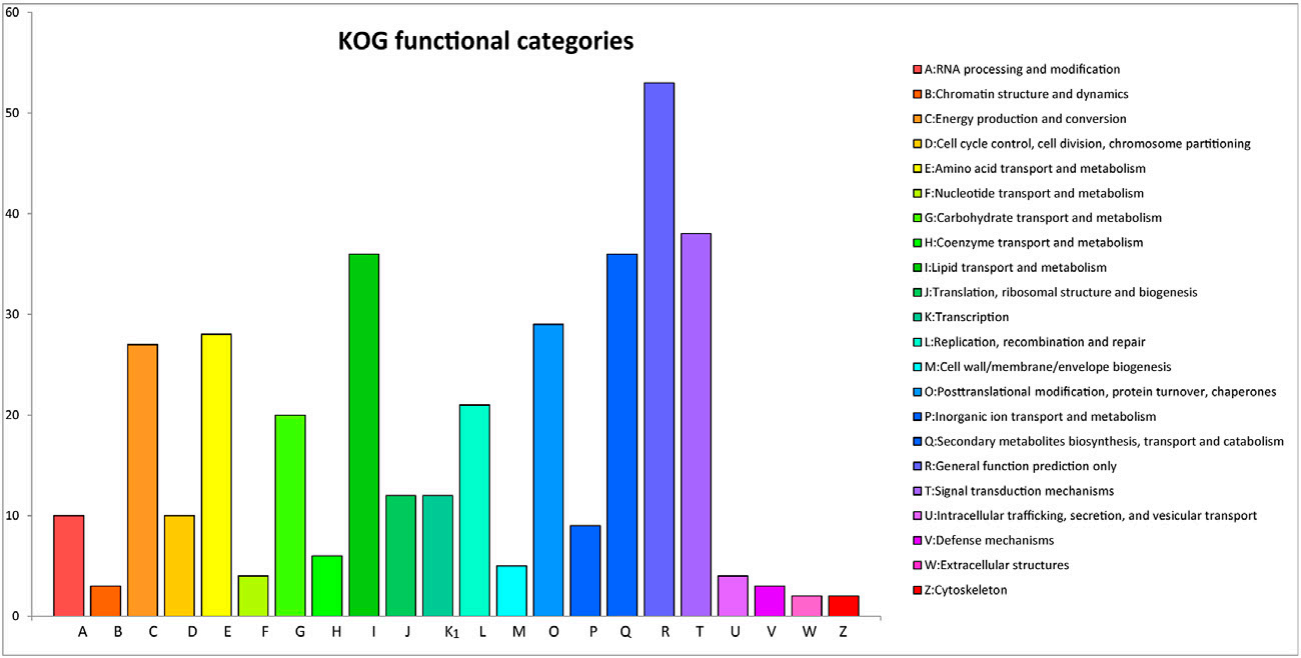
KOG classification.

**FIGURE 6:**
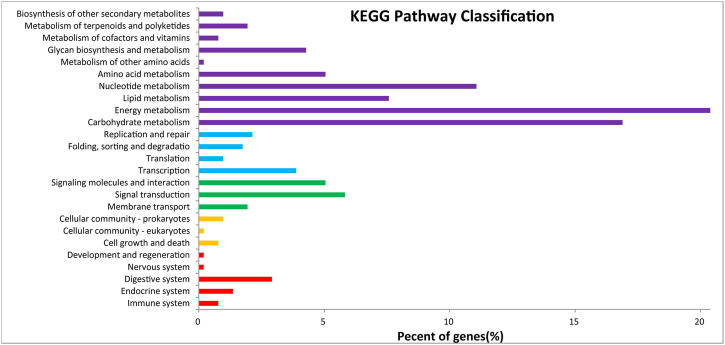
KEGG classification.

After GO annotation, the annotated genes were classified according to three GO categories: biological process, cellular component, and molecular function. A total of 336 annotated genes were obtained by GO annotation, accounting for 52.74% of the total number of genes (Figure 4). According to the annotations in the KOG database, 257 annotated genes were divided into 22 functional categories (Figure 5). KEGG analysis annotated 387 genes into 275 pathways, encompassing metabolism, genetic information processing, environmental information processing, cellular process, and organismal systems (Figure 6). At the same time, 441 of the 637 protein encoding genes were annotated by Blastp against *Arabidopsis*, rice, and *poplar* genomes, of which 383 genes were annotated into the *Arabidopsis* genome, 401 genes into the rice genome, and 359 genes into the *Populus* genome.

### 3.5 SSR Locus Analysis

**FIGURE 7:**
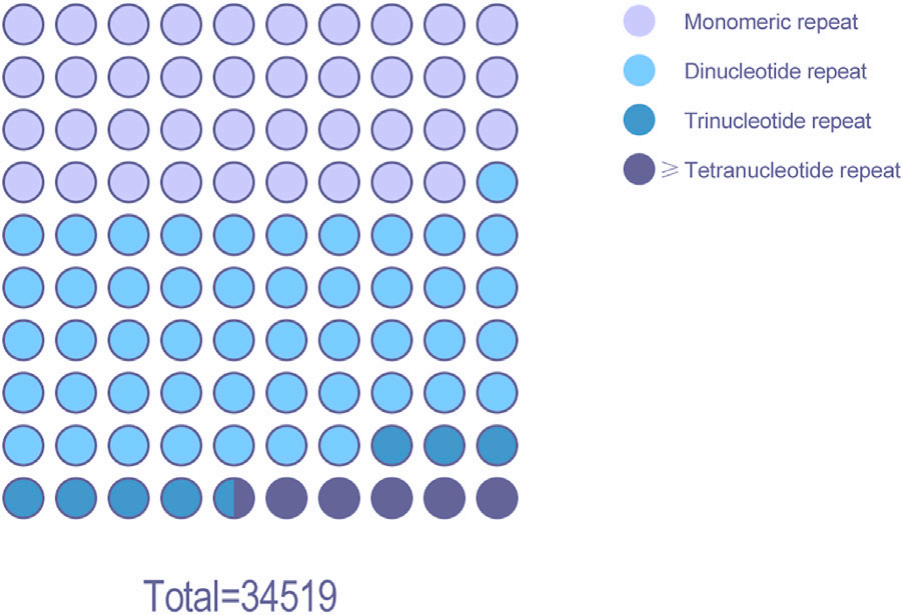
Percentagedot plot analysis of SSR locus statistics using BAC data.

The MISA script was used to analyze the BAC data of Chinese fir (Figure 7). A total of 34,519 SSR loci were searched, the proportion of dinucleotide repeats sites was the highest (16,604), accounting for 48.1% of the SSR loci. There were 13,444 single nucleotide repeat and 2,552 trinucleotide repeats, accounting for 38.9% and 7.4% of the SSR loci, respectively. Approximately 1,919 sites consisted of four or more nucleotide repeats, accounting for 5.6% of the SSR loci. Dinucleotide nucleotide repeats are the predominant SSRs in the Chinese fir BAC library.

### 3.6 Sequence identification

**FIGURE 8:**
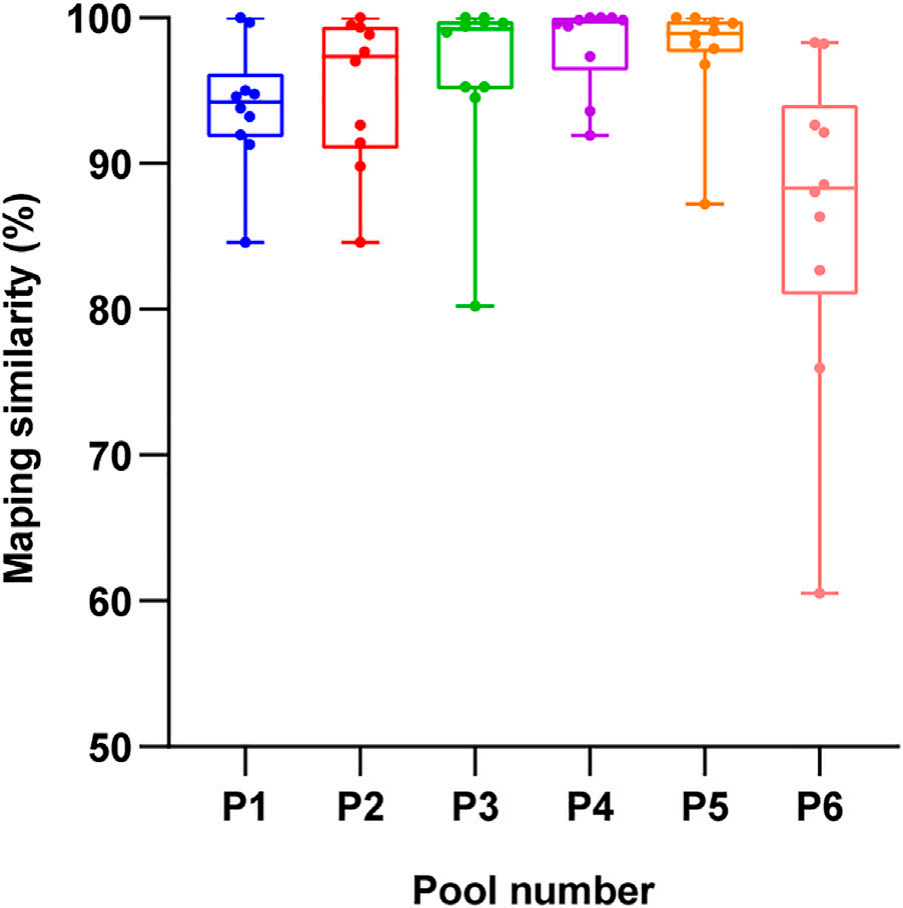
The fragment matching rate insix pools.

To confirm the accuracy of the BAC spliced sequence, the DNA of *Cunninghamia lanceolata* was extracted, the assembly sequences of pools 1 to 6 were used as target sequence. A total of 60 pairs of random primers (Supplementary Table S1) were designed and used to amplify the target sequences between 300 bp and 800 bp. Then, the results of Sanger sequencing were compared to the assembly file, and the matching rate was calculated. As shown in Figure 8, the fragment had a good matching rate in all 6 pools, and the matching rate reached 93.90%, 95.09%, 96.30%, 98.04%, 96.70%, and 85.47% in pools 1, 2, 3, 4, 5, and 6, respectively. These findings indicate that the BAC library is relatively accurate.

## 4 Conclusion and Discussion

It is difficult to extract high-molecular weight DNA. Phenolic and terpenoid compounds of secondary metabolites of *C. lanceolata* are easy to combine with nuclear DNA and precipitate, which is not conducive to enzyme digestion. At the same time, chloroplast DNA contamination will also affect BAC quality. In this study, β-mercaptoethanol and Triton X-100 were added to the eluent to extract high molecular-weight DNA. Triton X-100 is a mild surfactant that can increase the permeability of plasma membrane, dissolve lipids, and remove membrane systems. Chloroplast and mitochondria are organelles with well-developed membrane systems that can be well removed by Triton X-100, and it is easy to obtain high purity nuclear DNA from Chinese fir.

Tree genomes usually have high heterozygosity, and thus it is difficult to assemble BAC data using second and third generation sequencing data. Compared with Illumina platform data, PacBio platform sequencing data has some advantages, but the error rate is relatively high, and it is difficult to improve the quality due to its own sequencing technology. Therefore, when splicing BAC data, Illumina was selected as the benchmark, and PacBio data with longer reads were used as auxiliary. In the early stage of assembly, 50 BACs were randomly sequenced to verify the accuracy of high-throughput sequencing.

The accuracy of the reference sequence largely influences subsequent applications of the library. In this study, we designed 60 pairs of random primers with the lengths between 500 and 800 bp according to the reference sequence constructed by BAC library, and found that the correct rate of base is high. This shows that these reference sequences are reliable and essential for subsequent use of the BAC library.

Compared with the genomic characteristics of coniferous species, the genome of *C. lanceolata* is also large and complex. The genes that 500 BACs can mine are <1% of the whole genome. A total of 637 protein-coding genes were annotated using Spring and Phytozome databases. At present, the genomic resources of *C. lanceolata* are scarce. The establishment of a BAC library provides insights into its evolution and guide ongoing efforts to produce a reference genome sequence for *C. lanceolata*.

## Data Availability

The original of this study has been submitted to the NCBI database with ID is PRJNA803953. All data generated or analysed during this study are included in the article and Supplementary Material, other inquiries can be directed to the corresponding authors.
